# Monolithic MXene Aerogels Encapsulated Phase Change Composites with Superior Photothermal Conversion and Storage Capability

**DOI:** 10.3390/nano13101661

**Published:** 2023-05-17

**Authors:** Yan Wang, Fuqiang Wang, Changrui Shi, Hongsheng Dong, Yongchen Song, Jiafei Zhao, Zheng Ling

**Affiliations:** 1 Key Laboratory of Ocean Energy Utilization and Energy Conservation of Ministry of Education, School of Energy & Power Engineering, Dalian University of Technology, Dalian 116024, China; 2 Dalian Institute of Chemical Physics, Chinese Academy of Sciences, Dalian 116023, China

**Keywords:** MXene, phase change materials, solar energy utilization, photo-thermal conversion, aerogel

## Abstract

The inherently intermittent feature of solar energy requires reliable energy conversion and storage systems for utilizing the most abundant solar energy. Phase change materials are potential solutions to store a large amount of heat produced by solar light. However, few of the phase change materials have the ability to efficiently convert solar energy into heat; additionally, phase change materials need to be encapsulated in porous substrates for enhancing their leaking resistance and photo-to-thermal performance. In this work, monolithic MXene aerogels, fabricated by Al^3+^ cross-linking and freeze-drying, were used as the encapsulation and photothermal materials. The composites phase change materials of MXene/polyethylene glycol can be made with a large polyethylene glycol loading above 90 wt% with the maximum of 97 wt%, owing to the large porosity of MXene aerogels. The low content of MXene has a limited impact on the phase transition temperature and enthalpy of polyethylene glycol, with an enthalpy retention rate ranging from 89.2 to 96.5% for 90–97 wt% polyethylene glycol loadings. MXene aerogels greatly improve the leaking resistance of polyethylene glycol above its melting point of 60 °C, even at 100 °C. The composites phase change materials also show outstanding cycling stability for 500 cycles of heat storage and release, retaining 97.7% of the heat storage capability. The optimized composite phase change material has a solar energy utilization of 93.5%, being superior to most of the reported results. Our strategy produces promising composite phase change materials for solar energy utilization using the MXene aerogels as the encapsulation and photothermal materials.

## 1. Introduction

Solar energy, the zero-cost and most abundant renewable energy, has attracted growing attention due to society tackling dramatically increased CO_2_ emissions [1]. However, solar energy is inherently intermittent and, therefore, needs efficient and reliable systems for energy conversion and storage. Phase change materials (PCMs) can store large amounts of latent heat during phase transition [2,3]. Nevertheless, PCMs must be encapsulated for their practical utilization as the encapsulation in a shell material protects PCMs from leaking and increases specific surface areas to enhance heat transfer [2]. Additionally, most of the PCMs, particularly the organic ones, lack the ability to convert solar energy, resulting in low solar energy utilization efficiency. Therefore, it is highly desired to encapsulate PCMs into proper substrates for the conversion and storage of solar energy at a high efficiency. In recent years, porous substrate materials have become very popular. Many researchers are interested in them, not only due to their extremely large porosity, which can load as much as PCM, but also as a supporting skeletons, which can improve the thermal conductivity of PCMs.

Zhao et al. [4]. prepared a shape-stability composite PCM, in which a porous titanium dioxide foam (PTF) was used as the package carrier material and polyethylene glycol (PEG) as the PCM. The enthaply of melting of the PCMs is 145.9 J/g, and the actual impregnation ratio reached 70%. Li et al. [5] fabricated the lignin-based carbon composite PCMs by vacuum impregnation with PEG and porous carbon material. The thermal conductivity of composite PCM was 0.5 W/(m K), which is 53.8% higher than that of pure PEG. This is because the supporting skeleton material has excellent thermal conductivity. Zhu et al. [6]. fabricated composites by vacuum impregnation of a natural rubber-expanded graphite skeleton material in polyethylene glycol. The composition exhibited a high thermal conductivity of 1.05 W/(m K) and a high phase change enthalpy of 144.11 J/g. These studies found that porous materials can use physical forces to confine the PCM in the pores and prevent its leakage.

MXene is a family of two-dimensional transition metal carbides/nitrides, with impressive thermal conductivity and an abundance of surface functional groups. Similar to graphene, this property was reported by many researchers. Alexander et al. [7] reported the measurement of the thermal conductivity of a suspended single-layer graphene. The single-layer graphene performed at 5300 W/(m K) in a room by method of confocal micro-Raman spectroscopy.This suggests that graphene is extremely good at heat conduction. Jin et al. [8] investigated the thermal conductivity based on graphene (GR) and GR/MXene. They found that the addition of graphene and MXene could significantly improve the overall thermal conductivity of the material. Additionally, the heat transfer efficiency of the GR/MXene nanofluids was almost identical to that of the GR nanofluids. This suggests that the addition of MXene could have a similar effect on the improvement of the overall thermal conductivity. Most importantly, it was reported that Ti_3_C_2_T_x_, one of the most common MXenes, has a photo-to-thermal conversion efficiency of 100% [9]. These properties make MXene a promising candidate for encapsulating PCMs.

It is always critical to design new structures to load as many PCMs as possible without leaking and sacrificing the energy storage capability of the PCMs [3,10,11]. Multi-layered MXene particles were used to accommodate polyethylene glycol (PEG) to improve the light absorption and photo-thermal conversion efficiency of the composite PCMs [12]. The stratergy of using the monolithic substrate accommodating PCMs has attracted increasing attention due to the superior advantages of increasing the thermal conductivity and preventing PCMs from leaking. Foam and aerogels are the most popular and widely used structures, owing to the large porosity and low density, which can avoid PCM leakage with limited scarification in PCM loading.

Mo et al. fabricated a Ti_3_C_2_T_x_-based MXene/polyvinyl alcohol foam skeleton to hold PEG, with composite PCMs having 94.6 wt% PEG loading and a high photo-thermal conversion efficiency being produced [13]. Sheng constructed the pomelo peel foam/MXene/PEG composites by a simple impregnation process. The loading percentage and stored energy were simultaneously enhanced [14]. Fang fabricated porous potatoes@MXene/PEG composite PCMs by a vacuum-impregnation method. The photo-thermal storage efficiencies reach 98.5% due to the localized surface plasmon resonance effect of the Ti_3_C_2_T_x_ nanosheets [15]. However, the composite PCMs leak as the PCM loading further increases. Wang et al. prepared PEG/MXene-cellulose aerogels using a one-step in situ encapsulation method via freeze-casting. The MXene-enhanced PCMs showed a photo-thermal conversion efficiency of 91.6%, but the thermal conductivity was only 0.35 W/(m K) [16]. Lin et al. [17] prepared aerogel by freeze-drying, completed by mixing the MXene dispersion with PEG uniformly. The actual melting enthalpy of the composite PCM is 167 J/g, and the photo-thermal conversion efficiency achieved 92.5%. Lu et al. [18] successfully prepared composite PCMs by filling PEG into the MXene aerogel skeleton by the vacuum impregnation technique. The experimental results showed that the enthalpy of melting was 131 J/g and the thermal conductivity achieved was 2 W/(m K). Shen et al. [19] fabricated the composite PCMs with high thermal stability using the one-step method. The thermal conductivity increased 23.7% compared with that of pure PEG. However, the composite PCM showed leakage under 150 °C for 30 min.

Despite these reported MXene-containing composites, PCMs have shown impressive performance; however, some problems remain to be solved. For example, the preparation process is complex; the loading of PCMs is low; and the photo-thermal conversion efficiency needs to be improved.

To solve these problems, we fabricated composite PCMs using the one-step method of freeze-drying. The loading of PEG achieved 97 wt%. The composites PCMs show outstanding cycling stability for 500 cycles of heat storage and release, retaining 97.7% of the heat storage capability. The optimized composite PCM has a solar energy utilization of 93.5%, which is superior to most of the reported results.

## 2. Experimental Section

### 2.1. Materials

High-purity Ti_3_AlC_2_ MAX phase powders (500 mesh) were purchased from Laizhou Kai Kai Ceramic Materials Co., Ltd. in Laizhou, Shandong Province, China. Concentrated hydrochloric acid (HCl) was purchased from Sinopharm Chemical Reagent Co., Ltd. (Shanghai, China). Lithium fluoride (LiF) was bought from Alfa Aesar Chemical Co., Ltd. (Shanghai, China). PEG6000 and aluminum chloride (AlCl_3_·H_2_O) were bought from the Macklin Biochemic Co., Ltd. (Shanghai, China). All the chemical agents were used as received. Deionized water (18.2 MΩ cm) was produced via reverse osmosis in our laboratory.

### 2.2. Preparation of Ti_3_C_2_T_x_ MXene Nanoflake Dispersions and Aerogels

MXene was etched using our previously reported method [20]. In a typical run, 1.8 g of Ti_3_AlC_2_ was added into the mixed solution of 36 mL HCl (9 mol/L) and 2.88 g of LiF and then stirred at 45 °C for 48 h. After the etching reaction, the as-obtained product was centrifuged and washed 5 to 6 times with deionized water until the pH reached 6. A total of 60 mL of deionized water was added to the washed solid precipitate. The delamination was conducted using tip sonication for 60 min in the ice water bath under flowing nitrogen. Finally, the delaminated Ti_3_C_2_T_x_ MXene nanoflake dispersion was obtained after centrifugation at 3500 rpm for 30 min. The concentration of the delaminated MXene dispersion was measured by weighting the MXene nanoflakes from 3–5 mL dispersion.

The porous MXene aerogels were prepared via ion cross-linking. In a typical run, AlCl_3_ aqueous solution (400 μL, 0.5 mol/L) was dropwise added into 5 mL MXene dispersions (10 mg/mL). The MXene nanosheets were quickly cross-linked by shaking the plastic bottle gently for 1 min, forming hydrogel. Then, the hydrogel was frozen for 2 h in a household refrigerator and freeze-dried for 48 h to obtain the desired MXene aerogel.

### 2.3. Preparation of PEG@MXene Composites

The composite PCMs were prepared by the physical impregnation of PEG6000 into the as-made MXene aerogels. Solid PEG powder was put on the top of the MXene aerogel and heated at 100 °C for 12 h in a vacuum oven to facilitate the complete impregnation of PEG6000. The compositions of PEG@MXene were tuned via controlling the MXene and PEG6000 mass ratios (MXene:PEG = 1:9, 1:19, 1:32) with the MXene mass being constant in all the composites. The as-made PEG6000/MXene composites were labeled as *x*P@M, where P stands for PEG6000, M stands for MXene aerogel, and *x* stands for the mass fraction of PEG6000.

### 2.4. Characterization

The microstructures of MXene aerogels and *x*P@M were analyzed by scanning electron microscopy (Hitachi High-Technologies Corporation, Tokyo, Japan, SEM, SU8200). The crystal structures were studied using an X-ray powder diffractometer (Bruker Corporation, Billerica, MA, USA, Bruker D8 Advanced, 40 kV, 40 mA, λ = 0.154 nm). The chemical composition was analyzed by a Raman spectrometer (HORIBA Jobin Yvon, Paris, France, LabRAM HR Evolution, HORIBA) using a 532 nm excitation laser and 1800 lines/mm grating. The thermal stability, phase change properties, and thermal conductivity were investigated by a thermogravimetric analyzer (TGA, SETSYS 16/18, SETARAM Instruments, Lyon, France), a differential scanning calorimetry (DSC, Discovery, TA, Newcastle, DE, USA), and a thermal constants analyzer (TPS2500, Hot Disk, Gothenburg, Sweden). The thermal cycle stability was evaluated using a home-made cycle device with accelerated heating and cooling rates [21]. The UV-Vis-NIR absorption spectra of the composites were measured using a Hitachi U-4100 Spectrophotometer. The photo-thermal conversion and heat energy storage experiments were carried out by using a photo-thermal conversion system, which included a simulated light source (94023A Solar SIM/94023A, Newport Corporation, Deere Avenue, Irvine, CA, USA), photo-thermal conversion device (including a foam insulation layer, aerogel, and plastic beaker), and infrared thermal imager (FOTRIC 224S, Shanghai Thermal Image Technology Co., Ltd., Shanghai, China).

Phase change enthalpy retention *R* is the ratio of the melting enthalpy of the composites to that of pure PEG6000, which is used to evaluate the thermal energy storage performance of PCMs. *R* was calculated according to Equation (1):

(1) $$R=\frac{\Delta H_{m-c}}{\Delta H_{m-p}}\times 100\%$$

where ∆*H_m−c_* (J/g) is the actual melting enthalpy of the composites and ∆*H_m−p_* (J/g) is the melting enthalpy of the pure PEG6000.

The leaking resistance was evaluated using the following procedure. PEG6000 and *x*P@Ms were placed at a constant temperature (25 °C, 40 °C, 60 °C, 80 °C, and 100 °C) for 10 min, and their morphological changes were recorded with a digital camera.

Equation (2) is proposed to evaluate the solar–thermal conversion and storage efficiency (*η*) of the *x*P@Ms.

(2) $$\eta =\frac{m\times \Delta H_{m}}{P\times s\times \Delta t}\times 100\%$$

where *m* (g) is the mass of the sample, Δ*H_m_* (J/g) is the melting enthalpy, *P* (mW/cm^2^) represents the irradiation intensity of the sunlight simulator, *s* (cm^2^) means the surface area of the sample, and Δ*t* (s) represents the light-driven phase transition time of the sample in the phase change process.

## 3. Results and Discussion

### 3.1. Morphology and Structures of MXene and PEG@MXene PCMs

**Figure 1:**
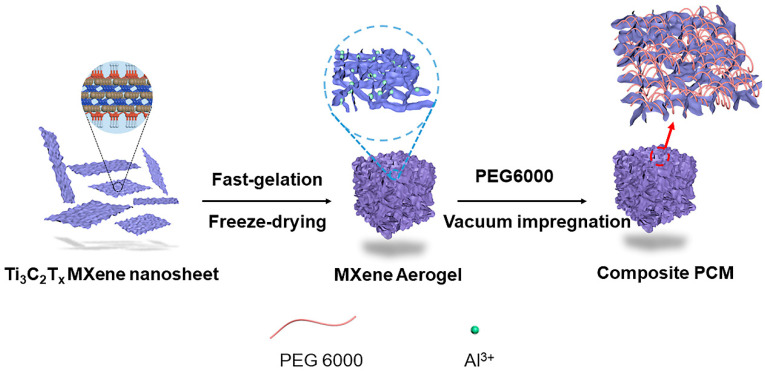
Schematic diagram of fabricating PEG6000@MXene composite PCMs.

The preparation process of *x*P@M is schematically shown in Figure 1. Dispersed Ti_3_C_2_T_x_ MXene nanosheets were produced via liquid-phase delamination. It has been demonstrated that high concentrations of nanosheets are critical for forming mechanically stable aerogels assembled from two-dimensional nanomaterials [22,23,24]. MXene dispersion with a high concentration of 10 mg/mL was used for fabricating MXene aerogels. Al^3+^ ions were utilized to cross-link Ti_3_C_2_T_x_ nanosheets via electrostatic interaction, inducing fast gelation to form MXene hydrogel. Monolithic MXene aerogel was produced after water in the hydrogel being removed by freeze-drying. The porous structure of MXene aerogel provides ideal room for accommodating PCMs and preventing them from leaking. The Ti_3_C_2_T_x_ MXene skeleton can turn solar energy into heat and transfer the heat to PCMs for storage. Melted PEG6000 infuses into the pores of MXene aerogel under vacuum. The MXene aerogel can absorb sunlight and convert the light into heat at a high efficiency, while PEG6000 can store the produced heat via solid–liquid phase change, making the *x*P@M composite a promising composite PCM for solar energy conversion and storage.

**Figure 2:**
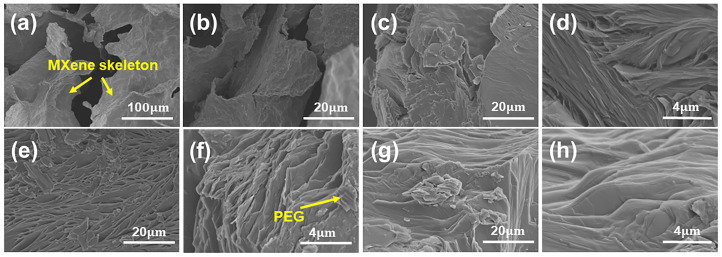
SEM images of (**a**,**b**) the MXene aerogel, (**c**,**d**) 90%P@M, (**e**,**f**) 95%P@M, and (**g**,**h**) 97%P@M.

The as-made MXene aerogel has hierarchic pore structures as indicated by the SEM images (Figure 2a,b). There are two kinds of pores formed by the MXene nanosheets stack and assembly. The cross-linked MXene nanosheets via Al^3+^ ions produce pores with feature sizes of around several tens of micrometers with obvious tortuosity, which is dramatically different from the pore structures produced via ice templates [25]. The ice templates usually result in orientated pores, which are easy for the leaking of melted PCMs and limit the loaded mass of PCMs. Due to the large porosity of aerogel, the as-made MXene aerogel can hold a large amount of PEG in the as-made composite PCMs. The *x*P@M composites with PEG6000 contents ranging from 90 wt% to 97 wt% were successfully produced, confirming the hierarchic pore structures being capable of accommodating large amounts of PCMs. Figure 2c–h show that the layered structures of MXene aerogels remain intact, and the interlayer and pores assembled from nanosheets are fully filled with PEG6000. The 97%P@M has the largest content of PEG6000 in the composite, which is larger than most of the reported results in the literature, as shown in Table S1 of the supporting information (SI). The largest PCM content can dramatically reduce the dead weight of the MXene matrix. It is worth noting that although the amount of MXene is as low as 3 wt%, it forms the monolithic and connected MXene network, benefiting the heat transfer.

**Figure 3:**
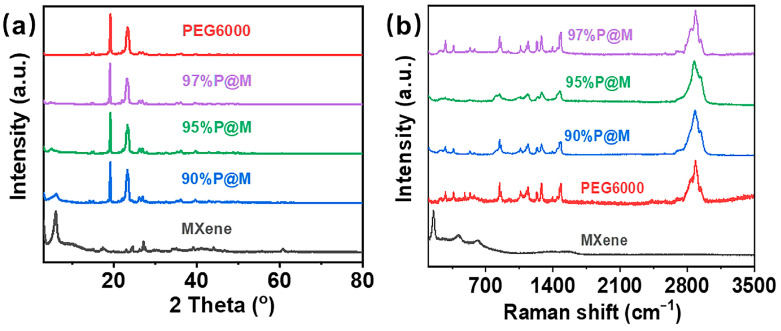
(**a**) XRD patterns and (**b**) Raman spectra of PEG6000, MXene aerogel, and the PEG6000/MXene composites with different PEG contents.

XRD patterns show that all the *x*P@M composites share similar diffraction peaks, indicating the similar crystal structures to that of pure PEG6000 (Figure 3a). The similar sharp characteristic peaks at 19.1° and 23.2° confirm that PEG6000 infused into MXene aerogel mainly exists in the form of crystalized PEG6000. The slightly reduced height and increased width of the characteristic peaks of the *x*P@M composites are caused by the impact of MXene nanosheets on the PEG6000 crystallization near the MXene surfaces. It is worth noting that the crystalline phase of Ti_3_C_2_T_x_ MXene at 5.9° becomes less with the increased PEG6000 loading and disappears in 97%P@M, because the amount of MXene gets lower and lower to 3 wt% in the *x*P@M composites. After infiltration of melted PEG6000, the interlayer distance between MXene nanosheets increases from 1.483 nm to 1.793 nm, indicating part of the PEG6000 is intercalated into the MXene nanosheets. The confined PEG6000 also contributes to its degraded crystalline phase mentioned above.

The chemical compositions of *x*P@M were analyzed by Raman spectroscopy as shown in Figure 3b. Raman peaks at 153 cm^−1^ and 420 cm^−1^ correspond to MXene and the peak at 2885 cm^−1^ belongs to PEG6000 [12,26]. Similar to the XRD results, the MXene network shows negligible Raman peaks as the PEG6000 content in *x*P@M increases. Since Raman spectroscopy is a surface sensitive characterization technology with limited detecting depth, the low amount of MXene surrounded by thick PEG6000 coating is hard for Raman spectroscopy to detect.

### 3.2. Phase Change Properties of xP@M PCMs

**Figure 4:**
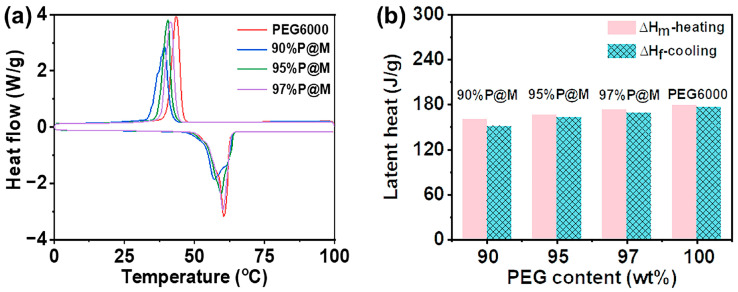
DSC thermogram of the composite PCMs with different PEG6000 contents, including (**a**) DSC curves and (**b**) the phase change enthalpy values.

Thermal energy storage performance is an essential characteristic of composite PCMs, so it is of great significance to study their heat storage and release performance. DSC was used to characterize the phase change behaviors of the *x*P@M composites and pure PEG6000 (Figure 4a). The *x*P@M composites were found to exhibit unimodal exothermic and endothermic behaviors similar to pure PEG6000.

It can be seen from the curves (Figure 4a) that the melting temperature of *x*P@M composites is below 60.4 °C, which is lower than that of pure PEG6000, indicating that the addition of supporting materials lowers the phase change temperature of PEG6000 [2]. The melting temperature of *x*P@Ms drops with the decreased contents of PEG6000. Figure 4b shows the enthalpy of transition for *x*P@Ms and PEG6000, and the specific data are shown in Table S2. As the PEG6000 loading in *x*P@Ms increases from 90 wt% to 97 wt%, the melting enthalpy of *x*P@Ms monotonically increases from 160.0 J/g for 90%P@M to 173.0 J/g for 97%P@M, approaching that of pure PEG6000 (179.3 J/g). The results confirm that using monolithic porous MXene aerogels can greatly improve PCMs loading and reduce the negative impact on the capacity in stored energy.

It can be seen from Table S2 that the melting and solidification temperatures of *x*P@M composites are slightly lower than those of pure PEG6000. With the decrease of PEG6000 content, the melting temperature decrease from 60.2 °C to 57.0 °C. The supporting materials could restrict the crystallization or thermodynamic behavior of PEG6000. Therefore, the phase change temperature and enthalpy of the *x*P@M could decrease [27]. Nevertheless, the melting enthalpy and freezing enthalpy still maintain a high level (more than 150.0 J/g). The melting enthalpy and solidification enthalpy of PEG6000 was slightly higher than that of the composite PCMs. Table S2 summarizes the PEG6000 content-dependent enthalpy and temperature of the phase change for *x*P@M composites and pure PEG6000. Through the calculation, the R of 90%P@M, 95%P@M, and 97%P@M are 89.2%, 92.9%, and 96.5%, respectively, which is higher than most of the reported PEG-based PCMs, as shown in Table 1.

### 3.3. Thermal and Cyclic Stability of PEG6000 and xP@M Composites

**Figure 5:**
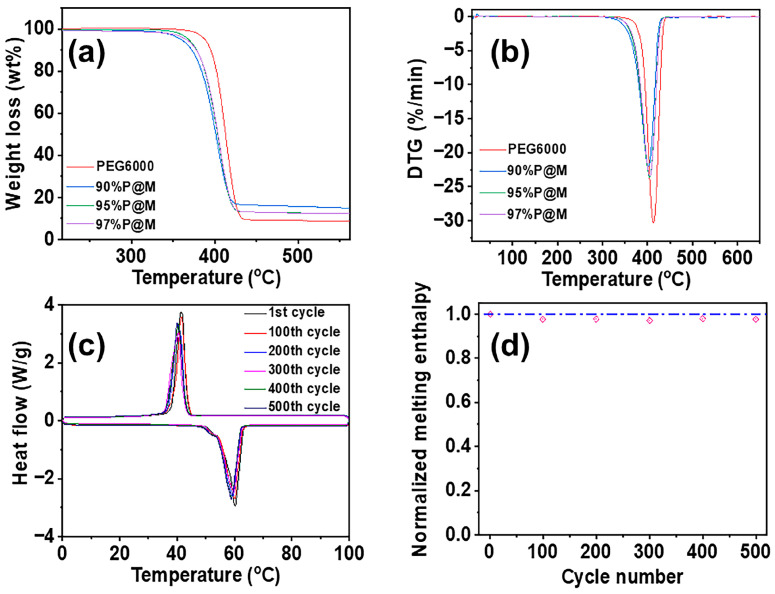
(**a**) TG and (**b**) DTG curves of PEG6000 and the composites with different PEG6000 contents in the atmosphere of nitrogen. (**c**) DSC curves of 95%P@M with different thermal cycle numbers. (**d**) Normalized melting enthalpy of 95%P@M with different thermal cycle numbers.

Thermal and cycling stabilities are the critical concerns for long-term utilization of PCMs. Thermogravimetric analysis was used to characterize the thermal stability of as-made *x*P@Ms. As shown in Figure 5a,b, PEG6000 and *x*P@Ms remain stable below 300 °C, which is enough for the materials used for solar light-to-heat conversion and storage. It can be found that MXene can promote the decomposition of PEG6000. The 90%P@M starts to decompose at 325 °C, which is lower than that of pure PEG6000 (360 °C) in the absence of MXene aerogels. MXene also plays a promoting role in the maximum decomposition temperature of the samples as indicated in Figure 5b. The 95%P@M shares a similar maximum decomposition temperature of 340 °C, which is 20 °C lower than that of pure PEG6000, confirming the promoting effect of the MXene aerogels in the thermal decomposition of PEG6000 above 300 °C.

Figure 5c shows the differential scanning calorimetric curve of 95%P@M at the first, 100th, 200th, 300th, 400th, and 500th thermal cycle. The change in the phase transition temperature of 95%P@M is negligible over 500 cycles, confirming the promising cycling stability. Besides the phase transition temperature, the change of melting enthalpy of 95%P@M is also negligible during 500 phase transition cycles as shown in Figure 5d. The normalized melting enthalpy, which corresponds to the retention rate, shows 0.005% decrease each cycle. The outstanding cycling stability indicates the heat energy storage and release is highly reversible over long-term cycling owing to the monolithic porous structure of the MXene aerogel.

**Figure 6:**
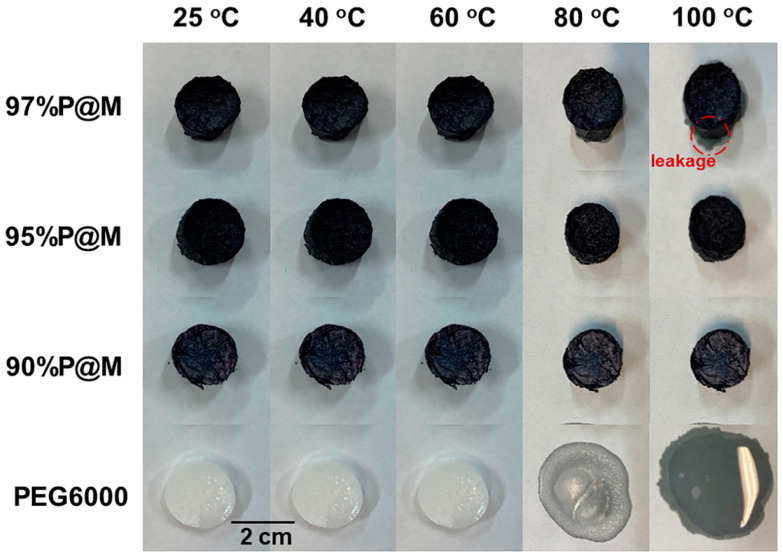
Comparison of the shape stabilization properties of PEG6000 and *x*P@M at different temperatures.

Morphologic stability is critical for the energy storage of PCMs based on the solid–liquid phase change. In order to evaluate the morphologic stability and leaking resistance of the composite aerogels, a leakage experiment was carried out. As shown in Figure 6, all samples retain their original shapes at 60 °C and below. When the temperature rises to 80 °C, partial melting and deformation of pure PEG6000 is obvious, and the filter paper is wetted by the melting PEG6000. However, there is no leakage observed in the *x*P@M composites. When the temperature is raised to 100 °C, PEG6000 in pure forms is completely melted and has a low viscosity. Surprisingly, 97%P@M shows slight leakage, while 90%P@M and 95%P@M show no signs of deformation and leakage, indicating the excellent structural stability and leakage resistance of the as-made *x*P@M composites. The impressive morphology stability and leaking resistance of the composites is attributed to the presence of the monolithic MXene aerogel network, which could provide the strong hydrogen bond interaction among MXene surface groups and hydroxyl groups of PEG6000 [35]. Capillary action of porous aerogels also plays an important role in preventing PEG6000 leaking [36,37]. The rigid porous structure, large porosity, ultralight density, and rich surface functional groups make the as-made MXene aerogels ideal substrates to loading PEG6000, producing composite PCMs with excellent structural stability and leaking resistance.

### 3.4. Photo-Thermal Conversion and Storage of xP@M

**Figure 7:**
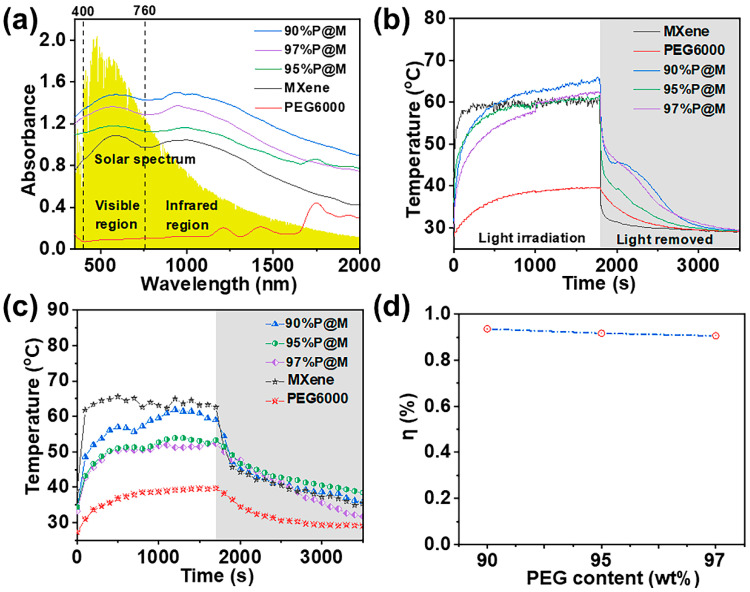
(**a**) Absorbance spectra of PEG6000 (diameter: 2 cm, thickness: 8 mm), MXene aerogel (diameter: 2.2 cm, thickness: 1.35 cm), 90%P@M (diameter: 2 cm, thickness: 1.15 cm), 95%P@M (diameter: 2 cm, thickness: 1.25 cm), and 97%P@M (diameter: 1.8 cm, thickness: 1.15 cm). Temperature evolution curves of the samples under the (**b**) simulated sunlight intensity of 100 mW/cm^2^ and (**c**) actual sunlight irradiation (T = 24 °C, 13:00–14:00, 30 May 2021, Dalian, China). (**d**) The photo-thermal storage efficiencies of the composites under the simulated sunlight irradiation.

MXene aerogel shows its typical light absorption spectrum in the UV-Visible-NIR region as shown in Figure 7a, exhibiting two enhanced absorption peaks in the both visible (around 592 cm^−1^) and near-infrared (around 998 cm^−1^) regions, which results in the localized surface plasmon resonance effect, facilitating the absorption and conversion of solar energy [12]. The two enhanced absorption peaks remain in *x*P@M, owing to the presence of the MXene aerogel as the skeleton in the composites. The enhanced light absorption capacity of *x*P@M compared to PEG6000 would boost energy conversion performance of the as-made composites.

MXene aerogel shows impressive solar light-to-heat performance as shown in Figure 7b. It takes only 98 s for MXene aerogel to reach 58 °C and stabilize at around 60 °C under the simulated solar light with a power density of 100 mW/cm^2^ (1 sun). The maximum heating rate is 0.8 °C/s as shown in Figure S1. The PEG6000 shows limited solar light-to-heat performance, resulting in a slow heating rate and a maximum temperature of 40 °C, which is much lower than that of MXene. It takes 113 s for 90%P@M and 95%P@M to rise from room temperature to 50 °C. In contrast, it takes 277 s for 97%P@M to rise to the same temperature, due to the increased PEG6000 content. After turning off the simulated sunlight, the temperature of MXene drops sharply to 34 °C and then gradually reaches room temperature, indicating the reversible heat storage and release.

Figure 7c shows the photo–thermal storage curves of the PEG6000 and *x*P@Ms under actual sunlight. When exposed to sunlight, the *x*P@Ms were rapidly heated up at a much faster rate than that of PEG6000 and reached a relatively stable temperature of 51.0–51.5 °C. The loading of PEG6000 in MXene aerogel delays the heating rate compared to pure MXene aerogel as shown in Figure 7c. However, 90%P@M shares a similar stable temperature at 56.0–57.0 °C, which is enough to initiate the phase transition of PEG6000, storing the solar energy via heat. As PEG6000 loading increases to 95 wt% and 97 wt%, the sunlight intensity is not enough to initiate the solid–liquid phase change by producing enough heat. This problem could be solved by choosing PCMs with lower phase-transition temperature. When the sun is blocked, the temperature drops rapidly first and then slowly to the ambient temperature. It is worth noting that *x*P@Ms show a much higher maximum temperature and reversible energy release ability (Figure 7c), making them promising candidates for the conversion and storage solar energy. It can be found that the photo-thermal conversion efficiencies (*η*) of the 90%P@M, 95%P@M, and 97%P@M are 93.5%, 91.6% and 90.7%, respectively (Figure 7d). Clearly, the photo-thermal conversion efficiency can be optimized by PEG6000 loading to fully utilize the monolithic MXene aerogels for photo-thermal conversion and heat transfer. Although 97%P@M has a high loading, it leaks slightly under 100 °C. In contrast, no leakage occurred in 90%P@M. It may be that the presence of MXene better limits the flow of PEG6000, so 90%P@M has excellent anti-leakage performance. The absorbance spectra of composite PCMs also show that 90%P@M has a stronger light absorbance than others, and photo-thermal conversion efficiency is highest among these samples. This may be because the thermal vibration of the molecular materials are more intense.

According to the experiment analysis, 90%P@M is suitable for solar energy conversion and heat storage.

## 4. Conclusions

MXene aerogels, fabricated by Al^3+^ cross-linking and freeze-drying, were used as the supporting substrates for loading PEG6000 to make ideal composite PCMs for solar energy conversion and storage. The monolithic porous structure of the MXene aerogel facilitates the PEG6000 loading, showing a maximum PEG6000 content of 97%. The MXene aerogel substrate has a limited impact on the phase transition temperature and enthalpy of PEG6000 at a loading above 90 wt%; the impact becomes negligible at a PEG6000 content of 97 wt%. The monolithic MXene aerogel as the support skeleton enables *x*P@Ms to have excellent morphological stability, leaking resistance, and cycling performance at 80 to 100 °C and over 500 cycles in heat storage and release. *x*P@Ms show strong solar light absorption capacity with enhanced absorption at visible and near-infrared regions and can spontaneously convert solar energy into heat energy. The 90%P@M has an outstanding photo-thermal storage efficiency of 93.5%, which shows a slight decrease as the PEG6000 content increased from 90 wt% to 97 wt%. This study demonstrated that monolithic MXene aerogel is a promising substrate for encapsulating PCMs for solar energy conversion and storage, owing to its outstanding photo-thermal properties, large porosity, and the stable structure. Our work has laid the foundation for the preparation of clay composite PCM, which have a lower cost.

In practical applications, the composite PCM prepared by us have great application prospects in the fields of solar energy storage, battery thermal management, wearable devices, and so on.

Although our work has prepared composite PCM with excellent photo-thermal conversion efficiency, the thermal conductivity still needs to be improved. It is critical to study the thermal conductivity, as it will increase the efficiency of the phase change storage. In the next phase of the research, our work will focus on the thermal discoloration of PCMs and the enhancement of thermal conductivity.

## Figures and Tables

**Table 1:** Comparison of enthalpy retention rate of different composite PCMs.

Composition of the Composite PCMs	Content of PEG	Retention of ΔH (R)	References and Year
*x*P@M	97.0%	96.5%	This work
Graphene aerogel/PEG2000	97.7%	91.0%	2020 [28]
CNT/graphene/PEG2000	98.8%	96.5%	2020 [29]
Ti_3_C_2_T_x_@PVA/PEG2000	92.3%	90.6%	2021 [13]
Ag/GNS/PEG6000	92.0%	89.6%	2019 [30]
HNT/Ag/PEG1000	45.0%	43.8%	2019 [31]
Ti_3_C_2_/PEG6000	80.0%	72.6%	2019 [12]
CF/SiO_2_/PEG6000	80.0%	67.3%	2018 [32]
SWCNTs/PEG6000	98.0%	97.8%	2017 [33]
Vermiculite/PEG4000	56.0%	54.2%	2018 [34]

## Data Availability

Not applicable.

## Supplementary Materials

The following supporting information can be downloaded at: https://www.mdpi.com/article/10.3390/nano13101661/s1, Figure S1: The heating rate of MXene aerogel; Table S1: Comparison of capability of MXene-based composite PCMs; Table S2: DSC results of different composite phase change materials. References [38,39,40,41,42,43,44,45,46,47,48,49,50,51,52,53,54] are cited in the supplementary materials.
