# Supplementary material for: Monolithic MXene Aerogels Encapsulated Phase Change Composites with Superior Photothermal Conversion and Storage Capability
Source: Nanomaterials (Basel). 2023 May 17;13(10):1661. doi: 10.3390/nano13101661 (PMC10222023; doi:10.3390/nano13101661)
Supplement: Supplementary file 1 [file nanomaterials-13-01661-s001.zip › nanomaterials-2381240-supplementary.pdf]

Monolithic MXene aerogels encapsulated phase change composites with superior photothermal conversion and storage capability

Yan Wang[1], Fuqiang Wang[1], Changrui Shi[1], Hongsheng Dong[2]*, Yongchen Song[1], Jiafei Zhao[1], Zheng Ling[1]*

[1]Key Laboratory of Ocean Energy Utilization and Energy Conservation of Ministry of Education, School of Energy & Power Engineering, Dalian University of Technology, Dalian 116024, China

[2]Dalian Institute of Chemical Physics, Chinese Academy of Sciences, Dalian 116023, China

*Corresponding authors. Tel.: +86 0411 84708015 E-mail addresses: dhsh2009@dicp.ac.cn (HS Dong), zling@dlut.edu.cn (Z Ling)

Table S1 Comparison of capability of MXene-based composite PCMs

Material	PEG content (wt%)	Phase-change temperature (ºC)	η (%)	Thermal conductivity (W/m K)	ΔH (J/g)	Ref.
$Ti_3C_2T_x$/PEG	97.0	60.0	93.5	NA	173.0	Our work
MXene/PEG	95.0	60.2	85.5	NA	167.7	[1]
MXene/PEG	85.0	59.0-63.0	94.5	NA	157.9	[2]
$Ti_3C_2T_x$/PEG	75.0	NA	95.0	1.7	93.4-119.1	[3]
MXene/PEG	97.0	60.0	NA	2.1	131.2	[4]
$Ti_3C_2T_x$/PEG	97.0	54.0	86.9	NA	142.1	[5]
SA@MXene/PEG	93.0	60.0	NA	NA	138.7	[6]
CNF@MXene@Fe_3O_4/PEG	NA	58.7	97.0	NA	151.0	[7]
PPF@MXene/PEG	96.2	62.0	NA	0.4	158.1	[8]
MXene-cellulose/PEG	90.0	56.0-60.0	91.6	NA	183.0	[9]
HDIT@MXene/PEG	NA	59.0	90.5	NA	127.8	[10]
MPMF/PEG	NA	60.4	NA	NA	186.2	[11]
MF@MXene/PEG	NA	57.3	92.7	NA	194.1	[12]
WPU@MXene/PEG	NA	60.5	NA	NA	154.6	[13]
MXene@PI/PEG	98.1	61.0	NA	NA	167.9	[14]
$Ti_3C_2T_x$@PVA/PEG	94.6	53.0	96.5	0.4	131.1	[15]

**$Ti_3C_2T_x$@PDA /PEG based polyurethane**	**NA**	**53.0**	**NA**	**NA**	**121.9-128.2**	[16]
**$Ti_3C_2T_x$@potato/PEG**	**82.1**	**60.0**	**98.5**	**NA**	**135.6**	[17]
**Bacterial cellulose@MXene/PEG**	**NA**	**NA**	**95.0-100.0**	**NA**	**192.2**	[18]
**GO@MXene/SA**	**90.0**	**70.4**	**93.0**	**NA**	**80.0**	[19]
**rGO@MXene/SA**	**NA**	**NA**	**90.0**	**1.2**	**168.2**	[20]
**MXene@CNTs@sodium alginate/TDA**	**91.0**	**NA**	**84.0**	**NA**	**217.8**	[21]
**CNF@MXene/erythritol**	**NA**	**NA**	**88.0**	**69.0**	**325.0**	[22]
**MXene/paraffin**	**NA**	**71.7**	**NA**	**0.2**	**110.7**	[23]
**Polyvinyl alcohol@MXene/paraffin**	**NA**	**71.3**	**NA**	**0.6**	**117.0**	[24]

(1) ΔH: Enthalpy of melting of aerogel

(2) η: Photothermal conversion efficiency; $\eta = \frac{m \times \Delta H_m}{P \times S \times \Delta t} \times 100\%$

m: The mass of aerogel; ΔH_m: Enthalpy of melting of aerogel; P: The power density of solar simulator; S: the aera of aerogel exposed to light; Δt: The time of phase change

(3) PPF: pomelo peel foam

(4) PVA: polyvinyl alcohol

(5) PDA: polymerized dopamine

Table S2 DSC results of different composite phase change materials

Sample	Phase transition	T(°C)		ΔH (J/g)		R (%)
		Heating	Cooling	Heating	Cooling	
PEG6000	Solid-liquid	60.4	43.4	179.3	176.3	100.0%
90%P@M	Solid-liquid	57.0	39.3	160.0	151.3	89.2%
95%P@M	Solid-liquid	59.5	40.6	166.6	162.7	92.9%
97%P@M	Solid-liquid	60.2	41.5	173.0	169.0	96.5%

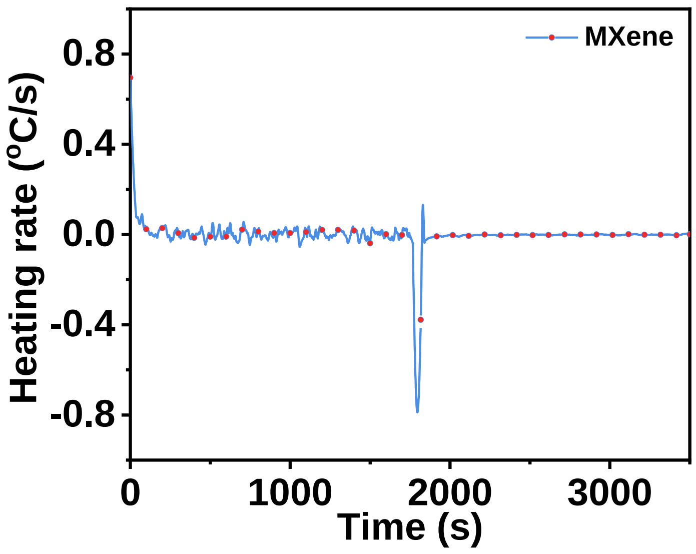

Figure S1. The heating rate of MXene aerogel

Reference：

[1] P. Lin, J. Xie, Y. He, X. Lu, W. Li, J. Fang, S. Yan, L. Zhang, X. Sheng, Y. Chen, MXene aerogel-based phase change materials toward solar energy conversion, Solar Energy Materials and Solar Cells 206 (2020) 110229. https://doi.org/10.1016/j.solmat.2019.110229.

[2] X. Fan, L. Liu, X. Jin, W. Wang, S. Zhang, B. Tang, MXene $Ti_3C_2T_x$ for phase change composite with superior photothermal storage capability, Journal of Materials Chemistry A 7(23) (2019) 14319-14327. https://doi.org/10.1039/c9ta03962g.

[3] J.L. Chen, Z.J. Mo, Y.Z. Chen, P.J. Mo, Z.Y. Hu, X. Chen, J. Yu, Z.F. Hao, X.L. Zeng, R. Sun, J.B. Xu, Highly Stable MXene-Based Phase Change Composites with Enhanced Thermal Conductivity and Photothermal Storage Capability, Acs Applied Energy Materials. https://doi.org/10.1021/acsaem.2c02140.

[4] X. Lu, H. Huang, X. Zhang, P. Lin, J. Huang, X. Sheng, L. Zhang, J.-p. Qu, Novel light-driven and electro-driven polyethylene glycol/two-dimensional MXene form-stable phase change material with enhanced thermal conductivity and electrical conductivity for thermal energy storage, Composites Part B: Engineering 177 (2019). https://doi.org/10.1016/j.compositesb.2019.107372.

[5] Y. Zhou, X. Wang, D. Sheng, X. Liu, Y. Yang, Light-driven PEG/$Ti_3C_2T_x$ form-stable phase change film for energy storage, Solar Energy Materials and Solar Cells 248 (2022) 112021. https://doi.org/10.1016/j.solmat.2022.112021.

[6] Y. Cao, W. Li, D. Huang, J. Zhang, P. Lin, L. Zhang, X. Sheng, Y. Chen, X. Lu, One-step construction of novel phase change composites supported by a biomass/MXene gel network for efficient thermal energy storage, Solar Energy Materials and Solar Cells 241 (2022). https://doi.org/10.1016/j.solmat.2022.111729.

[7] B. Quan, J. Wang, Y. Li, M. Sui, H. Xie, Z. Liu, H. Wu, X. Lu, Y. Tong, Cellulose nanofibrous/MXene aerogel encapsulated phase change composites with excellent thermal energy conversion and storage capacity, Energy 262 (2023). https://doi.org/10.1016/j.energy.2022.125505.

[8] X. Sheng, D. Dong, X. Lu, L. Zhang, Y. Chen, MXene-wrapped bio-based pomelo peel foam/polyethylene glycol composite phase change material with enhanced light-to-thermal conversion efficiency, thermal energy storage capability and thermal conductivity, Composites Part A: Applied Science and Manufacturing 138 (2020).

https://doi.org/10.1016/j.compositesa.2020.106067.

[9] H. Wang, Y. Deng, Y.L. Liu, F.Z. Wu, W.H. Wang, H.X. Jin, J.L. Zheng, J. Lei, In situ preparation of light-driven cellulose-Mxene aerogels based composite phase change materials with simultaneously enhanced light-to-heat conversion, heat transfer and heat storage, Composites Part a-Applied Science and Manufacturing 155 (2022). https://doi.org/10.1016/j.compositesa.2022.106853.

[10] S. Gong, Y. Ding, X. Li, S. Liu, H. Wu, X. Lu, J. Qu, Novel flexible polyurethane/MXene composites with sensitive solar thermal energy storage behavior, Composites Part A: Applied Science and Manufacturing 149 (2021). https://doi.org/10.1016/j.compositesa.2021.106505.

[11] Y. Du, H. Huang, X. Hu, S. Liu, X. Sheng, X. Li, X. Lu, J. Qu, Melamine foam/polyethylene glycol composite phase change material synergistically modified by polydopamine/MXene with enhanced solar-to-thermal conversion, Renewable Energy 171 (2021) 1-10. https://doi.org/10.1016/j.renene.2021.02.077.

[12] Y.-w. Shao, W.-w. Hu, M.-h. Gao, Y.-y. Xiao, T. Huang, N. Zhang, J.-h. Yang, X.-d. Qi, Y. Wang, Flexible MXene-coated melamine foam based phase change material composites for integrated solar-thermal energy conversion/storage, shape memory and thermal therapy functions, Composites Part A: Applied Science and Manufacturing 143 (2021). https://doi.org/10.1016/j.compositesa.2021.106291.

[13] W.-w. Hu, X.-y. Shi, M.-h. Gao, C.-h. Huang, T. Huang, N. Zhang, J.-h. Yang, X.-d. Qi, Y. Wang, Light-actuated shape memory and self-healing phase change composites supported by MXene/waterborne polyurethane aerogel for superior solar-thermal energy storage, Composites Communications 28 (2021). https://doi.org/10.1016/j.coco.2021.100980.

[14] Y.C.M.W.M.H.H.M.A.Y.E.L.Z.I.H.E.A.Y.C.M.H. Jintao Huang · Xinxin Sheng, <Flame-retardant and leakage-proof.pdf>, Advanced Composites and Hybrid Materials (2022). https://doi.org/10.1007/s42114-022-00504-4.

[15] Z. Mo, P. Mo, M. Yi, Z. Hu, G. Tan, M.S. Selim, Y. Chen, X. Chen, Z. Hao, X. Wei, $Ti_3C_2T_x$@Polyvinyl alcohol foam-Supported Phase Change Materials with Simultaneous Enhanced Thermal Conductivity and Solar-Thermal Conversion Performance, Solar Energy Materials and Solar Cells 219 (2021). https://doi.org/10.1016/j.solmat.2020.110813.

[16] X. Du, J. Qiu, S. Deng, Z. Du, X. Cheng, H. Wang, $Ti_3C_2T_x$@PDA-Integrated Polyurethane Phase Change Composites with Superior Solar-Thermal Conversion Efficiency and Improved Thermal Conductivity, ACS Sustainable Chemistry & Engineering 8(14) (2020) 5799-5806. https://doi.org/10.1021/acssuschemeng.0c01582.

[17] Y. Fang, S. Liu, X.L. Li, X.P. Hu, H. Wu, X. Lu, J.P. Qu, Biomass porous potatoes/MXene encapsulated PEG-based PCMs with improved photo-to-thermal conversion capability, Solar Energy Materials and Solar Cells 237 (2022). https://doi.org/10.1016/j.solmat.2021.111559.

[18] L. Tang, X. Zhao, C. Feng, L. Bai, J. Yang, R. Bao, Z. Liu, M. Yang, W. Yang, Bacterial cellulose/MXene hybrid aerogels for photodriven shape-stabilized composite phase change materials, Solar Energy Materials and Solar Cells 203 (2019). https://doi.org/10.1016/j.solmat.2019.110174.

[19] B. Yang, T. Zhang, J. Wang, J. Lv, Y. Zheng, Y. Zhang, Y. Wang, Novel properties of stearic acid / MXene - Graphene oxide

shape - Stabilized phase change material: Ascended phase transition temperature and hierarchical transition, Solar Energy Materials and Solar Cells 247 (2022). https://doi.org/10.1016/j.solmat.2022.111948.

[20] X. Wang, W. Yu, L. Wang, H. Xie, Vertical orientation graphene/MXene hybrid phase change materials with anisotropic properties, high enthalpy, and photothermal conversion, Science China Technological Sciences 65(4) (2022) 882-892. https://doi.org/10.1007/s11431-021-1997-4.

[21] X. Ye, Y. Ma, Z. Tian, H. Sun, Z. Zhu, J. Li, W. Liang, A. Li, Shape-stable MXene/sodium alginate/carbon nanotubes hybrid phase change material composites for efficient solar energy conversion and storage, Composites Science and Technology 230 (2022). https://doi.org/10.1016/j.compscitech.2022.109794.

[22] X. Du, J. Wang, L. Jin, S. Deng, Y. Dong, S. Lin, Dopamine-Decorated $Ti_{(3)}C_{(2)}T_{(x)}$ MXene/Cellulose Nanofiber Aerogels Supported Form-Stable Phase Change Composites with Superior Solar-Thermal Conversion Efficiency and Extremely High Thermal Storage Density, ACS Appl Mater Interfaces 14(13) (2022) 15225-15234. https://doi.org/10.1021/acsami.2c00117.

[23] N. Aslfattahi, R. Saidur, A. Arifutzzaman, R. Sadri, N. Bimbo, M.F.M. Sabri, P.A. Maughan, L. Bouscarrat, R.J. Dawson, S.M. Said, B.T. Goh, N.A.C. Sidik, Experimental investigation of energy storage properties and thermal conductivity of a novel organic phase change material/MXene as A new class of nanocomposites, Journal of Energy Storage 27 (2020). https://doi.org/10.1016/j.est.2019.101115.

[24] J. Zheng, Y. Deng, Y. Liu, F. Wu, W. Wang, H. Wang, S. Sun, J. Lu, Paraffin/polyvinyl alcohol/MXene flexible phase change composite films for thermal management applications, Chemical Engineering Journal 453 (2023). https://doi.org/10.1016/j.cej.2022.139727.
